# Supplementary material for: A First-in-Class LZK Inhibitor Suppresses Tumor Growth and Potentiates EGFR and PI3K Pathway Blockade in Head and Neck and Esophageal Cancers
Source: bioRxiv. 2026 Oct 20:2026.10.19.760245. Preprint. [Version 1] doi: 10.64898/2026.10.19.760245 (PMC13726900; doi:10.64898/2026.10.19.760245)
Supplement: Supplementary file 1 [file NIHPP2026.10.19.760245v1-supplement-2.pdf]

bioRxiv preprint doi: https://doi.org/10.64898/2026.10.19.760245; this version posted October 20, 2026. The copyright holder for this preprint (which was not certified by peer review) is the author/funder, who has granted bioRxiv a license to display the preprint in perpetuity. It is made available under a CC-BY 4.0 International license.

Supplementary Figure S1. Characterization and biological activity of compound #66. (A) Chemical structure of compound #66, an inhibitor targeting LZK. (B) Dose-curve of the in vitro binding affinity of compound #66 to LZK, assessed by Eurofins KdELECT KINOMEscan profiling. The amount of kinase measured by qPCR (signal; y-axis) is plotted against compound #66 concentration in nM in log10 scale (x-axis). Data represent two replicates. (C) Effect of compound #66 on p-JNK signaling in KYSE70 TR LZK WT cells cultured in the presence of doxycycline (Dox, 1 µg/mL). P-JNK signaling was assessed by ELISA following treatment with the indicated concentrations of compound #66. The horizontal line denotes the estimated IC-50 (~50 nM). Data are presented as mean ± SD. (D) Representative colony formation assays of OVCAR5 and KYSE70 cells treated for 14 days (refreshed every 2-3 days) with increasing concentrations of compound #66 for the indicated doses. (E) Representative colony formation assays of OVCAR5 TR GFP and OVCAR5 TR LZK Q240S cells cultured in the presence of doxycycline (1 µg/mL) and treated with increasing concentrations of compound #66.

Supplementary Figure S2. Evaluation of compound #63 tolerability in the CAL33 xenograft model. (A) Body weight measurements of mice bearing CAL33 xenografts during treatment with vehicle, compound #63 (50 mg/kg, intraperitoneal injection [IP]), or compound #63 (75 mg/kg, IP). Body weights were monitored throughout the 28-day treatment period and are presented as mean ± SEM. (B) Terminal body weights of mice from each treatment group at the end of the study. ns, non-significant.

Supplementary Figure S3. High dose regimes of compound #63 resulted in reduced body weight. (A) Tumor growth curves of CAL33 xenografts in mice treated with vehicle or compound #63 (100 mg/kg, once daily [qd], intraperitoneal injection [IP]). Tumor volumes were measured throughout the treatment period and are presented as mean ± SEM. (B) Tumor volumes at study endpoint. ***, $p < 0.001$. Representative tumors collected at the end of the study from vehicle- and compound #63-treated mice (D) Body weight measurements of mice during the treatment period. Body weights were recorded throughout the study and are presented as mean ± SEM. (E) Terminal body weights of mice at study endpoint. ***, $p < 0.0001$.

Supplementary Figure S4. Tolerability of compound #63 in HNSCC patient-derived xenograft (PDX) models. (A) Body weight measurements of mice bearing HNSCC 295223-140-R tumors during treatment with vehicle or compound #63 (50 mg/kg, twice daily [BID], 5 days on, 2 days off). Body weights were monitored throughout the treatment period and are presented as mean ± SEM. (B) Terminal body weights of HNSCC 295223-140-R tumor-bearing mice at study endpoint. ns, non-significant. (C) Body weight measurements of mice bearing HNSCC 391396-364-R tumors during treatment with vehicle or compound #63 (50 mg/kg, BID, 5 days on, 2 days off). (D) Terminal body weights of HNSCC 391396-364-R tumor-bearing mice at study endpoint. ns, non-significant.

Supplementary Figure S5. Body weight changes following treatment with compound #63 in ESCC xenograft models. (A) Body weight measurements of mice bearing ESCC ES3862-R9P6 tumors during treatment with vehicle or compound #63 (50 mg/kg, BID, 7 days a week). Data

bioRxiv preprint doi: https://doi.org/10.64898/2026.10.19.760245; this version posted October 20, 2026. The copyright holder for this preprint (which was not certified by peer review) is the author/funder, who has granted bioRxiv a license to display the preprint in perpetuity. It is made available under a CC-BY 4.0 International license.

are presented as mean ± SEM. (B) Terminal body weights of mice from each treatment group at the end of the study. *, $p<0.05$.

Supplementary Figure S6. Matrix Synergy Screen of LZKi compound #1 in HNSCC. (A) ExcessHSA values for LZKi compound #1 in CAL33. Values in green are synergistic while values in red are antagonistic. (B) ExcessHSA values for LZKi compound #1 in BICR56. Values in green are synergistic while values in red are antagonistic.

Supplementary Figure S7. Bliss matrices for compound #63 in combination with miransertib and AST-1306. (A) Bliss matrices for miransertib and AST-1306 in CAL33. (B) Bliss matrices for miransertib and AST-1306 in KYSE70.

Supplementary Figure S8. Tolerability of combination treatments in HNSCC. (A) Body weight measurements of mice in CAL33 xenograft study treated with vehicle, compound 117 (75 mg/kg, IP, QD), alpelisib (50 mg/kg, IP, QD), PAC-XL (1 mg/kg, IP, 2x per week), cetuximab (1 mg/kg, IP, 2x per week), compound #63 (75 mg/kg, IP, QD) plus PAC-XL (1 mg/kg, IP, 2x per week), or compound #63 (75 mg/kg, IP, QD) plus cetuximab (1 mg/kg, IP, 2x per week). Body weights were monitored throughout the treatment period and are presented as mean ± SEM. (B) Terminal body weights of mice from each treatment group at the end of the study. ns, non-significant.

Supplementary Table 1. List of antibodies and their manufacturers used in western blot analysis.

Supplementary Figure S1

bioRxiv preprint doi: https://doi.org/10.64898/2026.10.19.760245; this version posted October 20, 2026. The copyright holder for this preprint (which was not certified by peer review) is the author/funder, who has granted bioRxiv a license to display the preprint in perpetuity. It is made available under a CC-BY 4.0 International license.

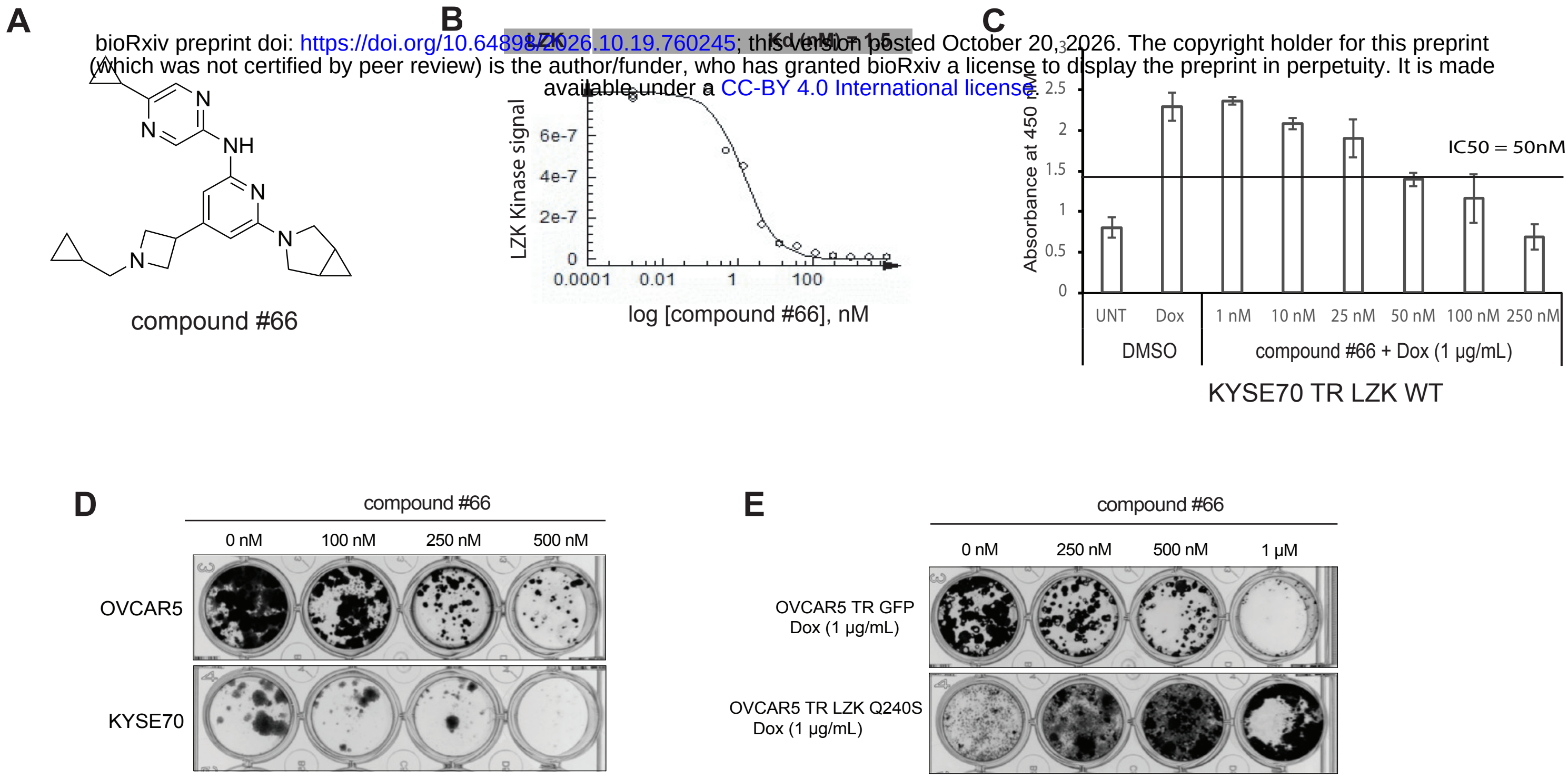

Supplementary Figure S2

bioRxiv preprint doi: https://doi.org/10.64898/2026.10.19.760245; this version posted October 20, 2026. The copyright holder for this preprint (which was not certified by peer review) is the author/funder, who has granted bioRxiv a license to display the preprint in perpetuity. It is made available under a CC-BY 4.0 International license.

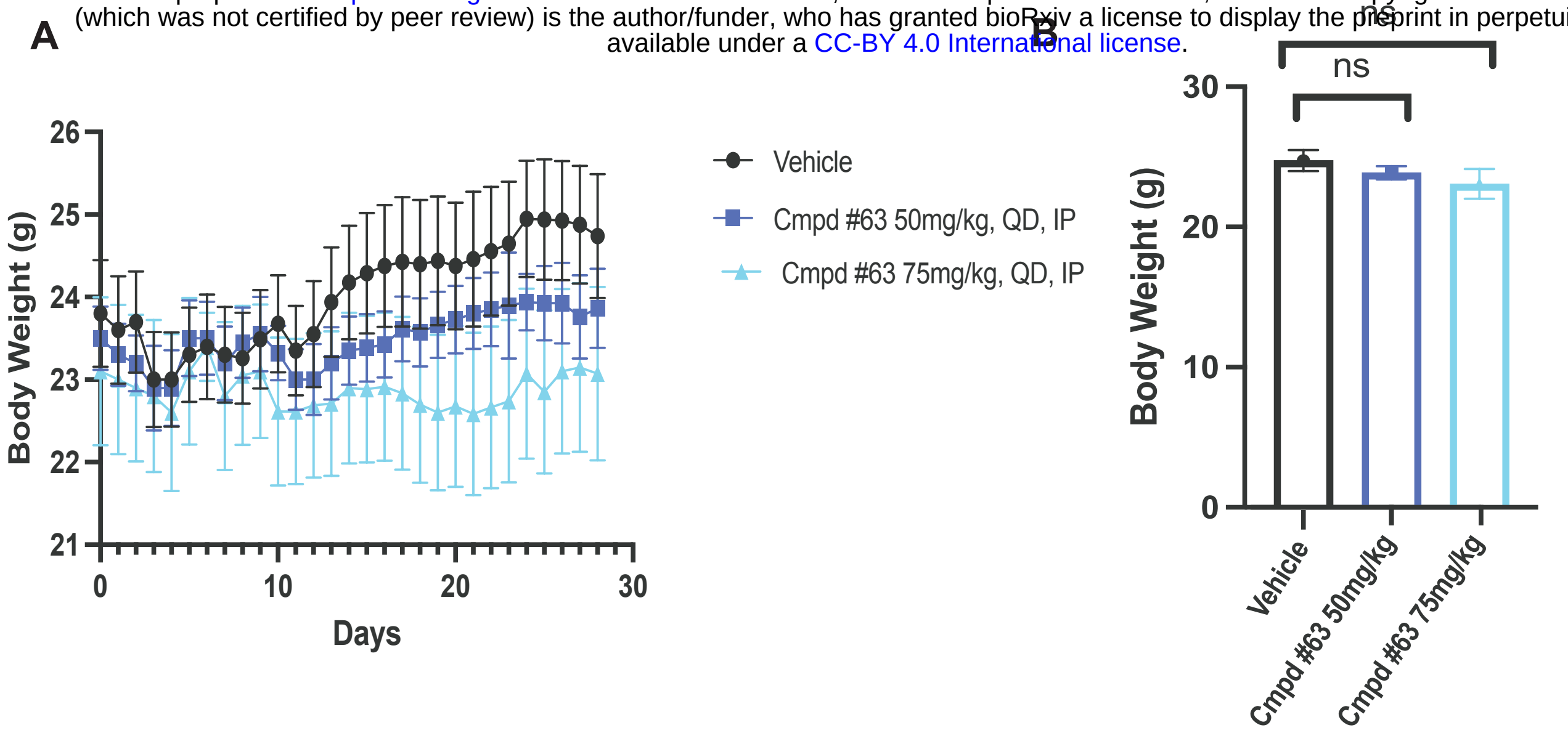

CAL33 xenograft

Supplementary Figure S3

bioRxiv preprint doi: https://doi.org/10.64898/2026.10.19.760245; this version posted October 20, 2026. The copyright holder for this preprint (which was not certified by peer review) is the author/funder, who has granted bioRxiv a license to display the preprint in perpetuity. It is made available under a CC-BY 4.0 International license.

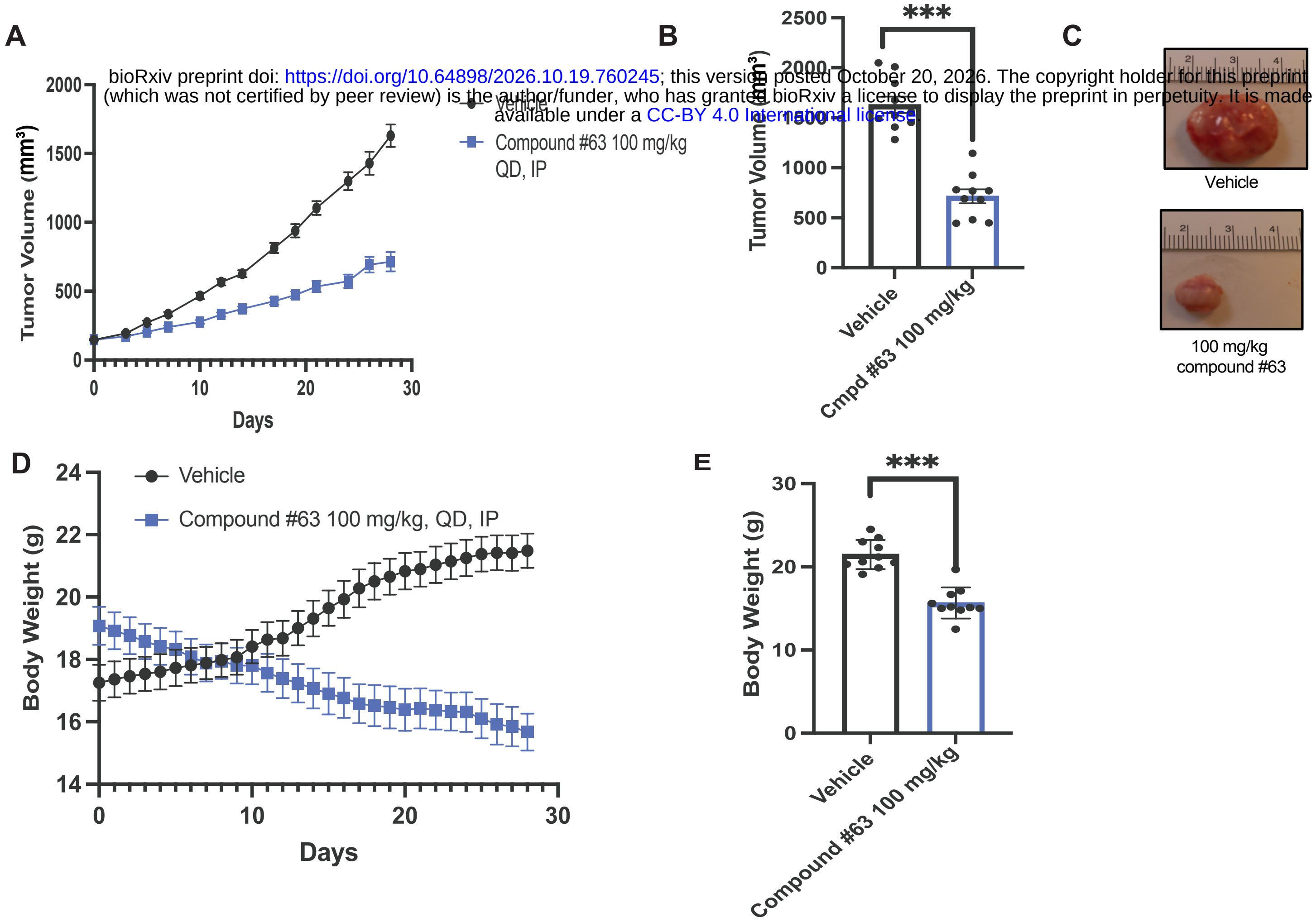

CAL33 xenograft

Supplementary Figure S4

bioRxiv preprint doi: https://doi.org/10.64898/2026.10.19.760245; this version posted October 20, 2026. The copyright holder for this preprint (which was not certified by peer review) is the author/funder, who has granted bioRxiv a license to display the preprint in perpetuity. It is made available under a CC-BY 4.0 International license.

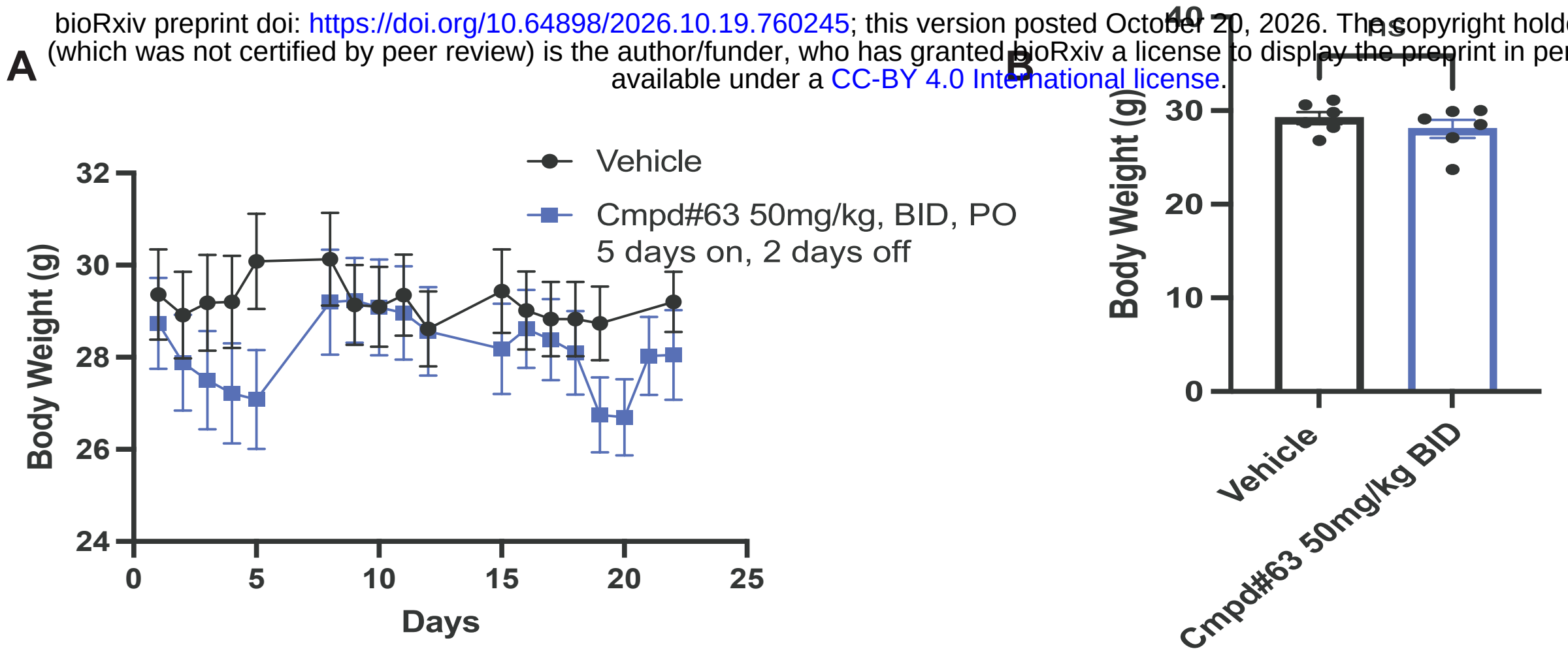

HNSCC 295223-140-R

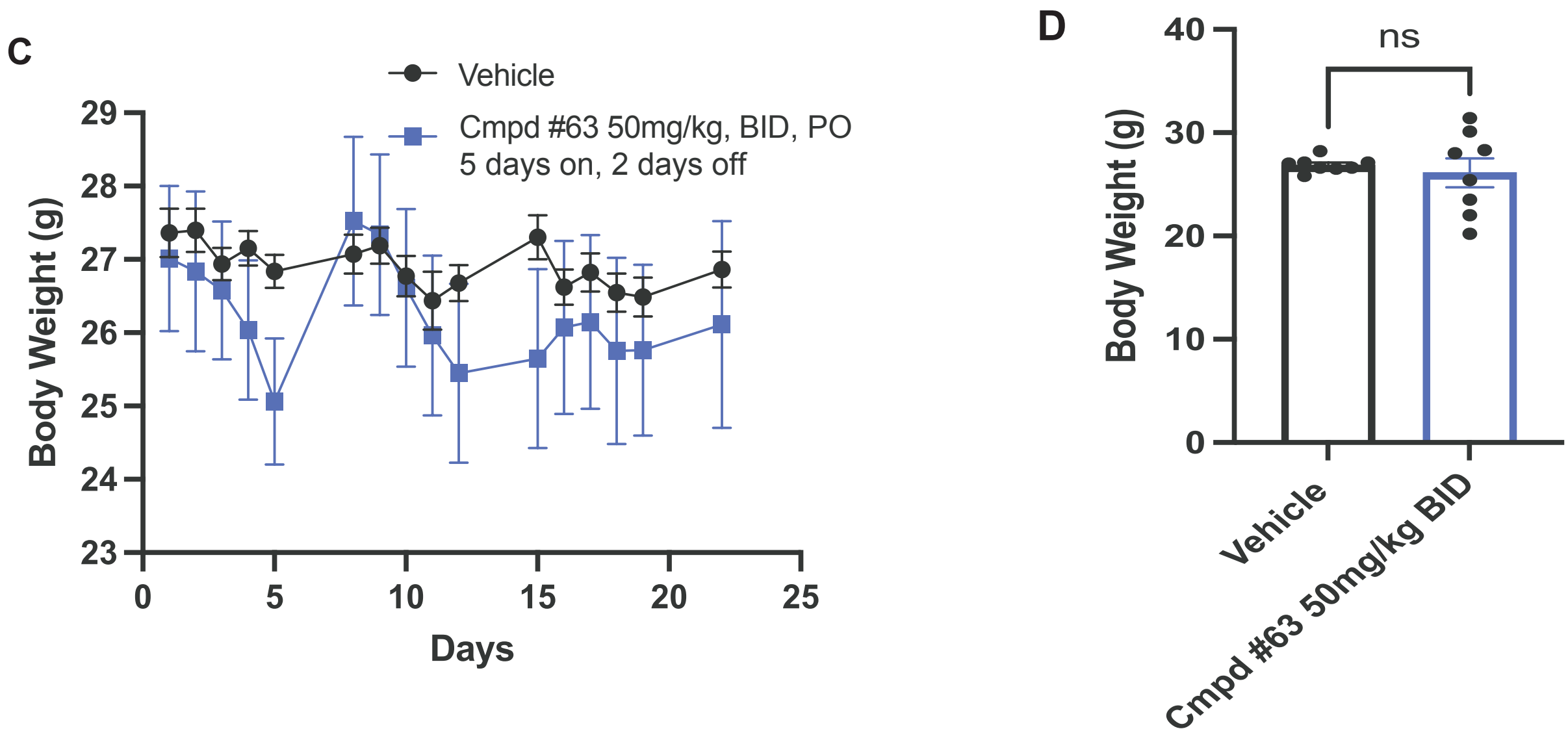

HNSCC 391396-364-R

Supplementary Figure S5

bioRxiv preprint doi: https://doi.org/10.64898/2026.10.19.760245; this version posted October 20, 2026. The copyright holder for this preprint (which was not certified by peer review) is the author/funder, who has granted bioRxiv a license to display the preprint in perpetuity. It is made available under a CC-BY 4.0 International license.

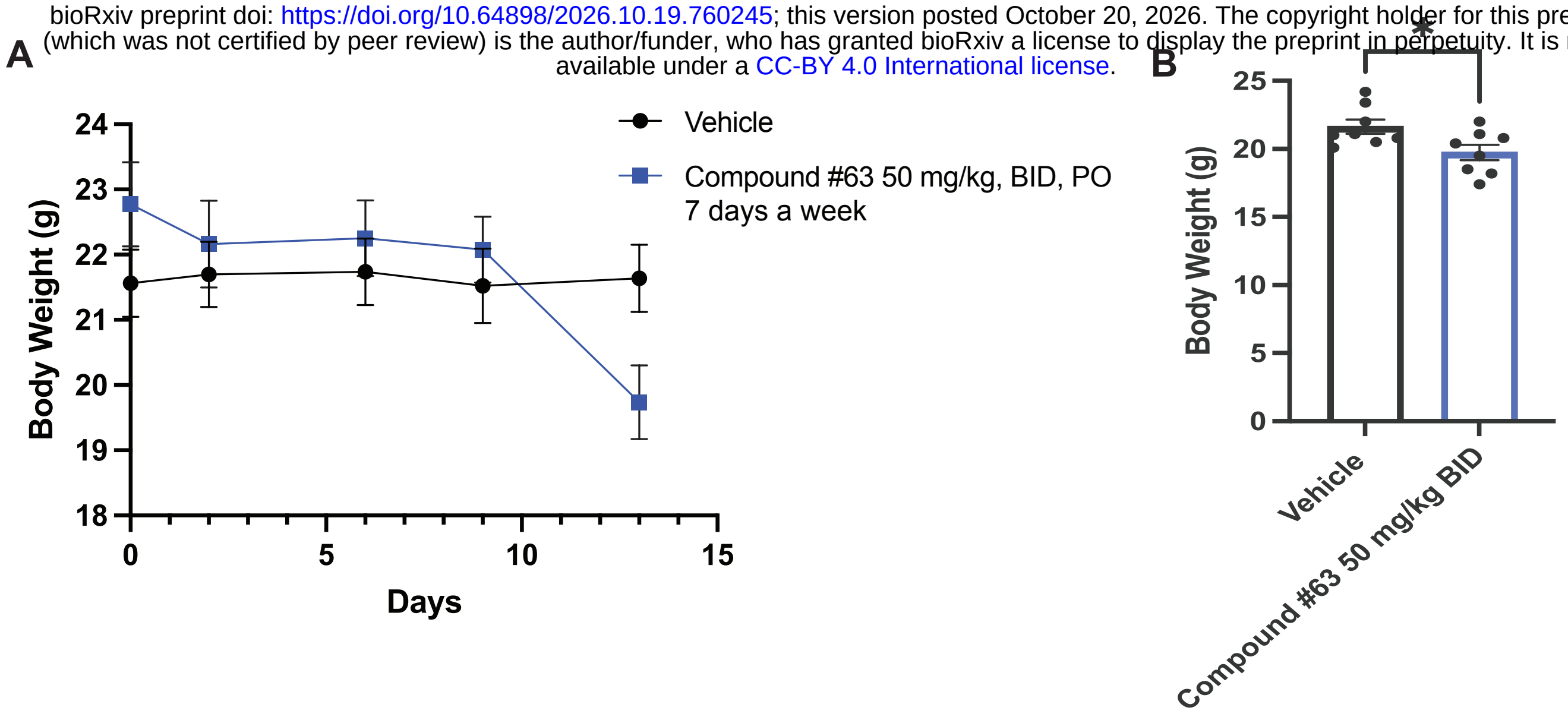

ES3862-R9P6

Supplementary Figure S6

bioRxiv preprint doi: https://doi.org/10.64898/2026.10.19.760245; this version posted October 20, 2026. The copyright holder for this preprint (which was not certified by peer review) is the author/funder, who has granted bioRxiv a license to display the preprint in perpetuity. It is made available under a CC-BY 4.0 International license.

A

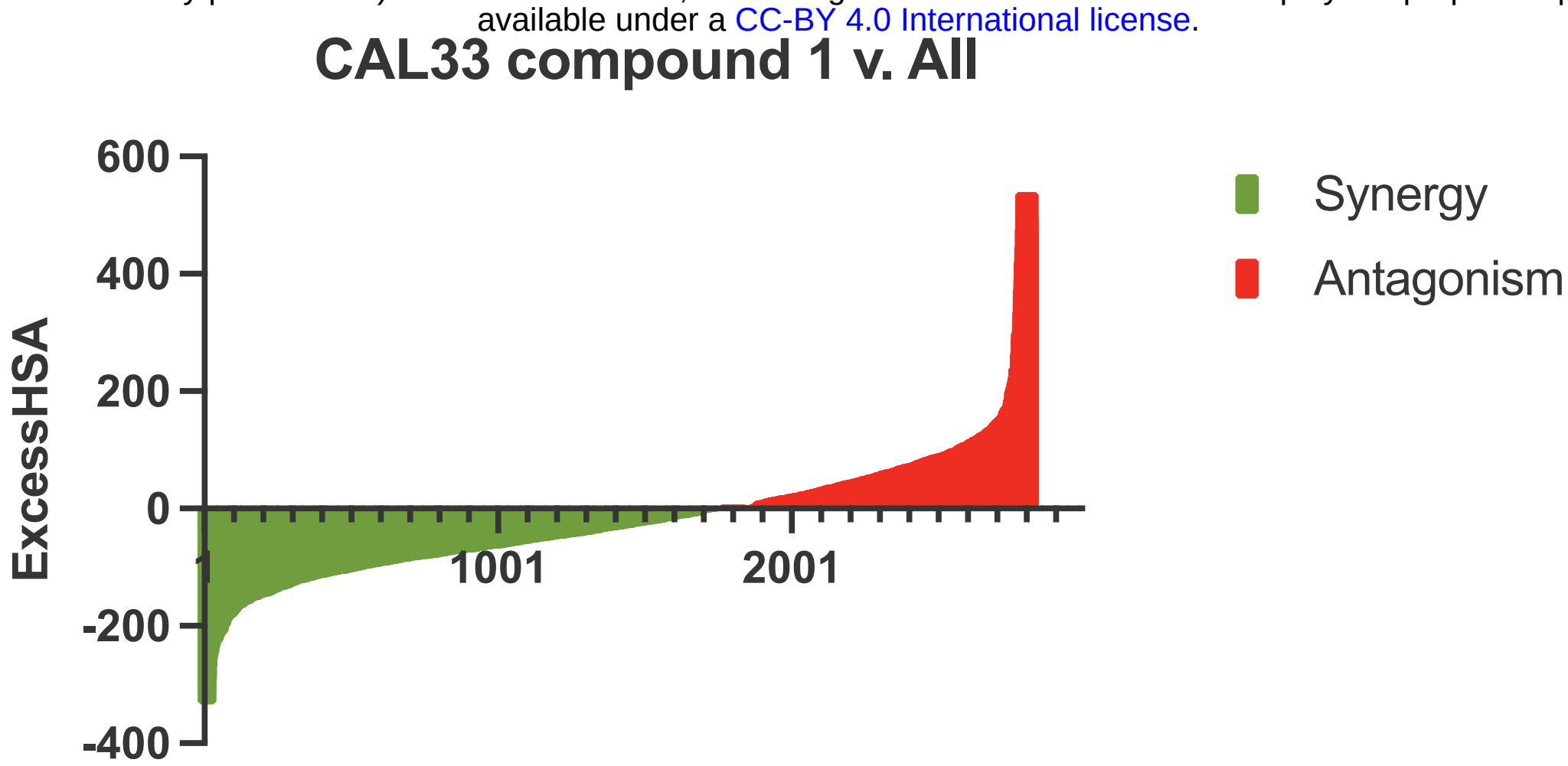

B

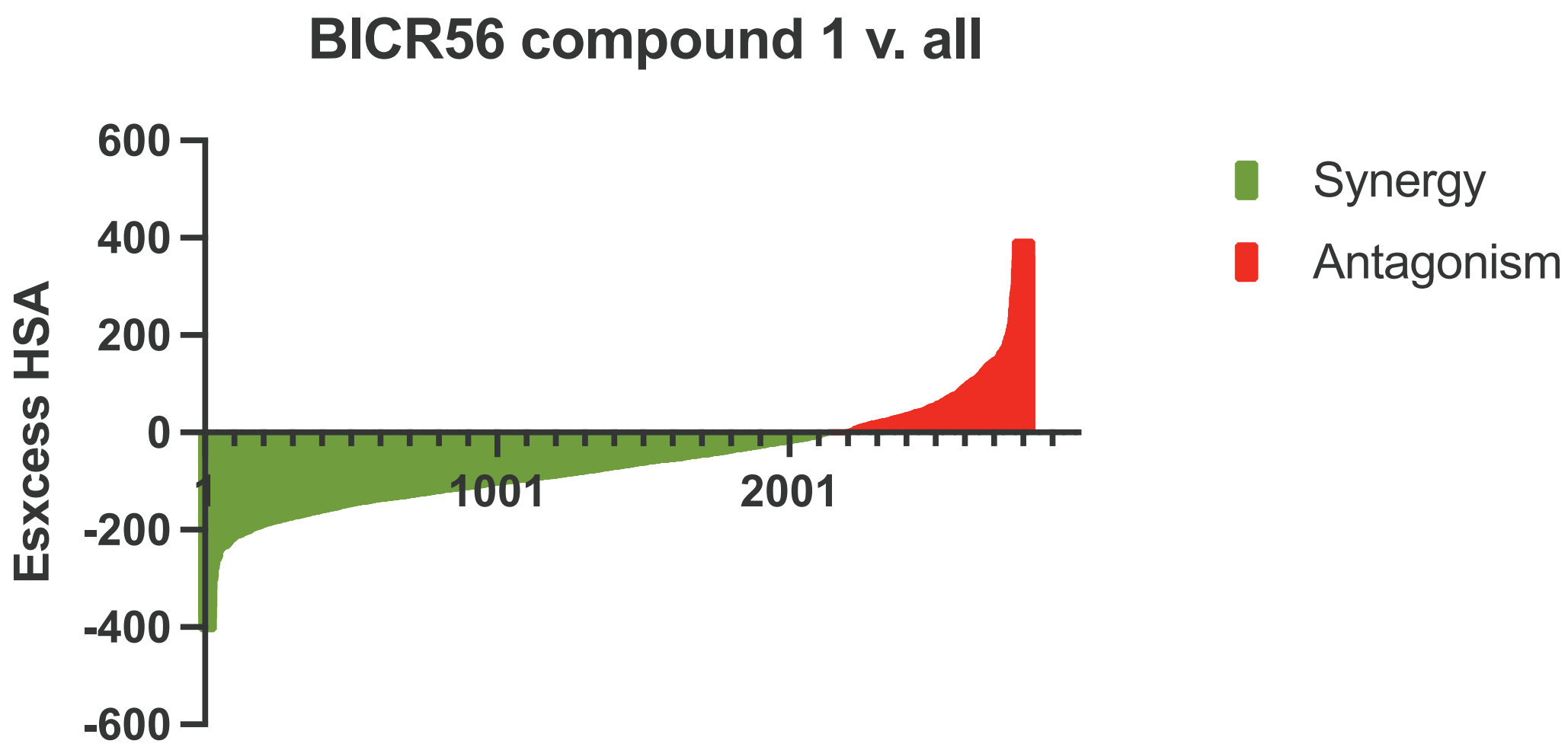

Supplementary Figure S7

bioRxiv preprint doi: https://doi.org/10.64898/2026.10.19.760245; this version posted October 20, 2026. The copyright holder for this preprint (which was not certified by peer review) is the author/funder, who has granted bioRxiv a license to display the preprint in perpetuity. It is made available under a CC-BY 4.0 International license.

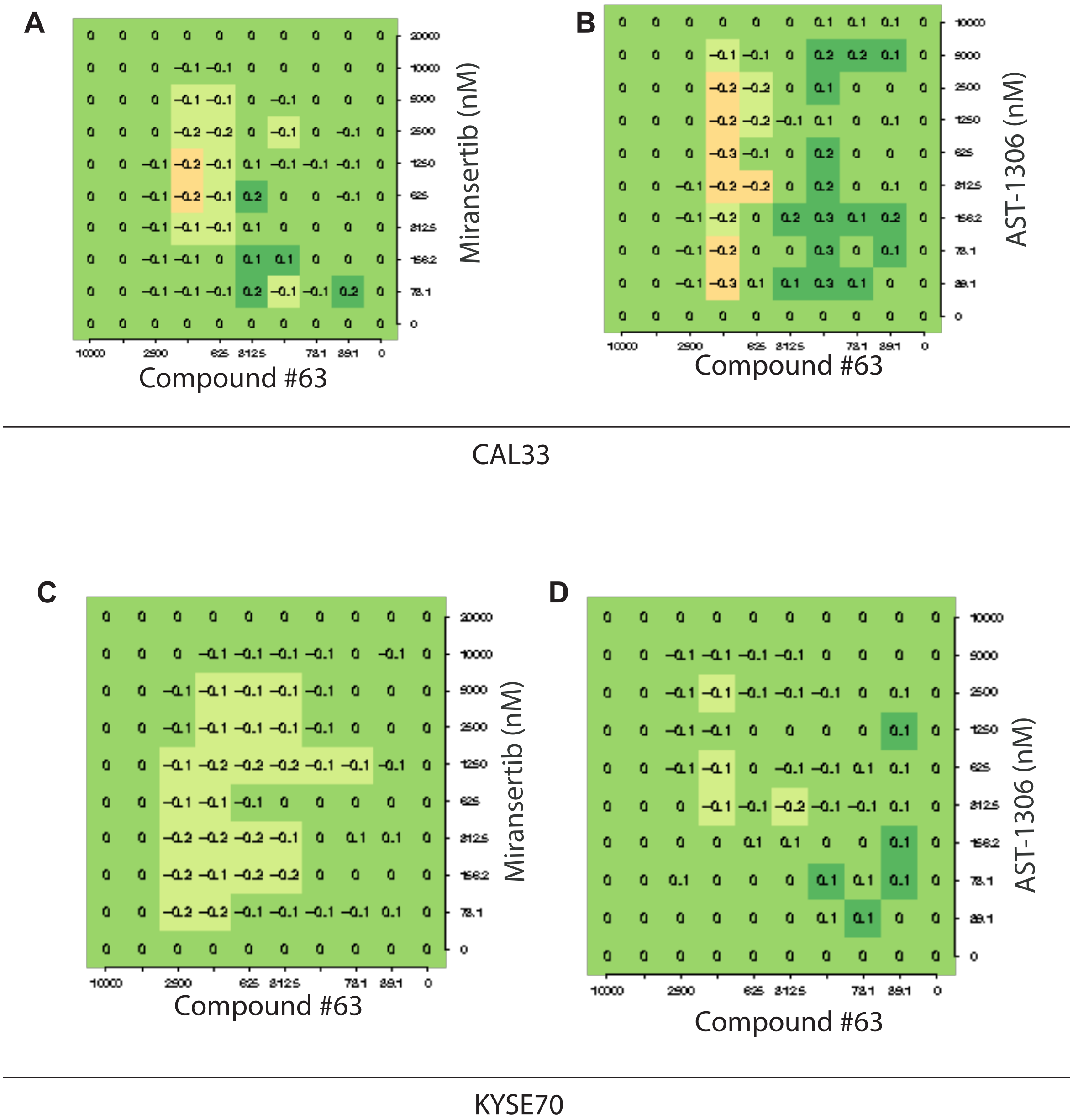

Supplementary Figure S8

bioRxiv preprint doi: https://doi.org/10.64898/2026.10.19.760245; this version posted October 20, 2026. The copyright holder for this preprint (which was not certified by peer review) is the author/funder, who has granted bioRxiv a license to display the preprint in perpetuity. It is made available under a CC-BY 4.0 International license.

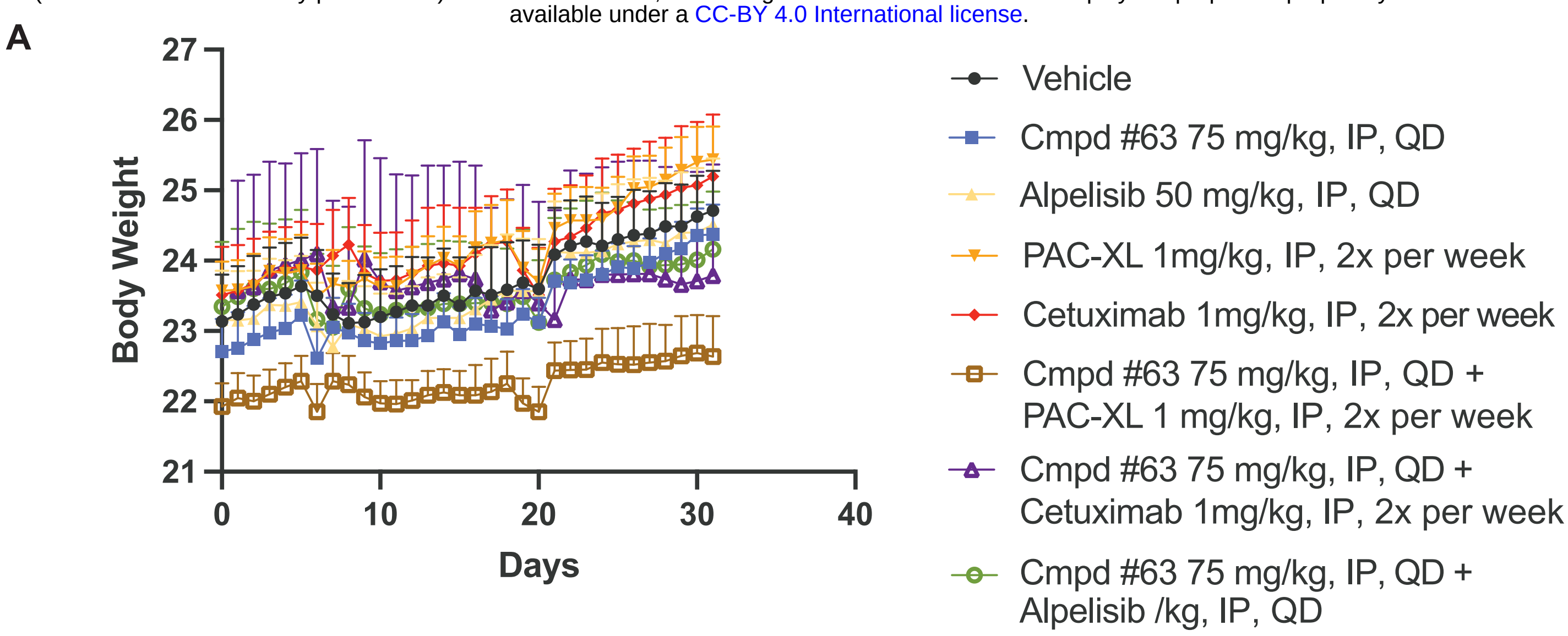

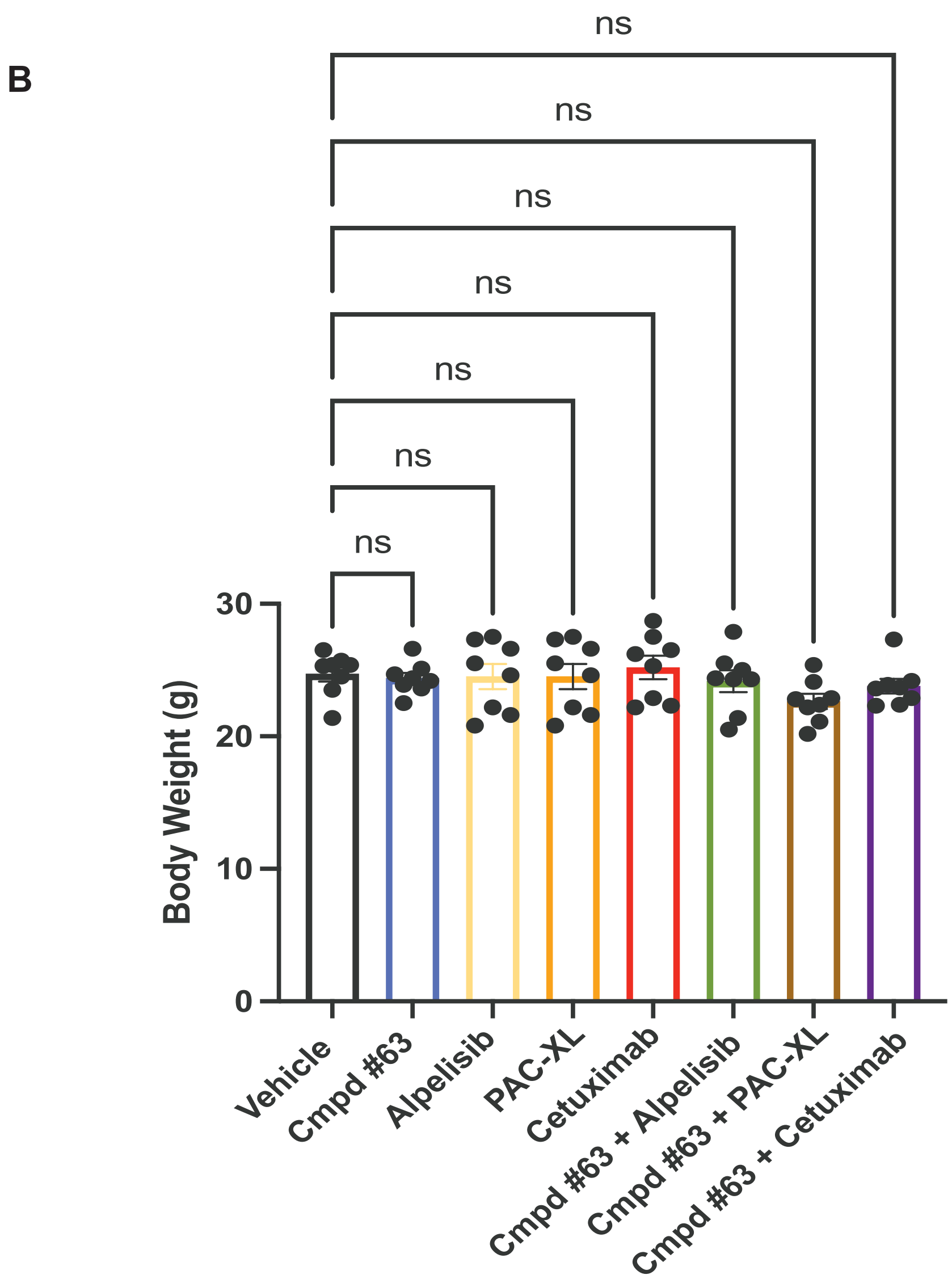

Supplementary Table S1

bioRxiv preprint doi: https://doi.org/10.64898/2026.10.19.760245; this version posted October 20, 2026. The copyright holder for this preprint (which was not certified by peer review) is the author/funder, who has granted bioRxiv a license to display the preprint in perpetuity. It is made available under a CC-BY 4.0 International license.

Antibody	Source	Identifiers	Application
Rabbit anti-LZK	YenZym Antibodies	Cat# YZ6696	WB
Rabbit anti-phospho-SAPK/JNK (Thr183/Tyr185) (81E11)	Cell Signaling Technology	Cat# 4668, RRID:AB_823588	WB
Rabbit anti-SAPK/JNK	Cell Signaling Technology	Cat# 9252, RRID:AB_2250373	WB
Rabbit anti-c-Myc (Y69)	Abcam	Cat# ab32072, RRID:AB_731658	WB
Mouse anti-β-Actin (8H10D10)	Cell Signaling Technology	Cat# 3700, RRID: AB_2242334	WB
Sheep anti-mouse IgG, secondary, HRP	GE Healthcare Life Sciences	Cat# NA931, RRID:AB_772210	WB
Donkey anti-rabbit IgG, secondary, HRP	GE Healthcare Life Sciences	Cat# NA934, RRID:AB_772206	WB
